# Targeting Endogenous Tau in a Human Tau Seeded Tauopathy Models Inhibits Tau Spread

**DOI:** 10.1002/alz.089470

**Published:** 2025-01-03

**Authors:** Hong Xu

**Affiliations:** ^1^ Center for Neurodegenerative Disease Research, PHILADELPHIA, PA, USA; University of Pennsylvania, Philadelphia, PA USA

## Abstract

**Background:**

Neurodegenerative diseases with the presence of tau pathology account for 90% of dementia in human patients, the most common of which is Alzheimer’s disease (AD). Although therapeutic approaches targeting tau and tau pathology are still under development, it remains unclear how tau targeting antibodies can inhibit the development of tau pathology.

**Method:**

We hypothesize tau antibodies enter neurons and inhibit the seeding of tau pathology without direct interaction to the pathogenic tau seeds. To tackle this, we designed a study using the human tau seeds‐induced sporadic spreading model of tauopathy to evaluate the effect of a mouse tau‐specific antibody (mTau8) on tau spreading *in vitro* and *in vivo*.

**Result:**

Our results show that mTau8 enters the cerebrospinal fluid by bypassing the blood‐brain barrier and effectively decreases AD‐tau‐seeded tau pathology in the wild‐type mouse and cell models. Moreover, the analysis of the spread of tau pathology *in vivo* and live imaging study *in vitro* suggest a mechanism that is independent of neutralizing the pathogenic tau seeds.

**Conclusion:**

Our findings provide a mechanistic view of antibody‐based tau therapy for treating tauopathies.

